# Supplementary material for: Natural and hybrid immunity following four COVID-19 waves: A prospective cohort study of mothers in South Africa
Source: eClinicalMedicine. 2022 Sep 17;53:101655. doi: 10.1016/j.eclinm.2022.101655 (PMC9481335; doi:10.1016/j.eclinm.2022.101655)
Supplement: Supplementary file 1 [file mmc1.docx]

**Supplementary material**

**Table S1. Characteristics of participants by seroprevalence in first wave**

|  | **All** | **SARS-CoV2-S seropositive** | **SARS-CoV2-S seronegative** | **p-value** |
| --- | --- | --- | --- | --- |
| **Maternal characteristics** | n=339 | n=176 (51·9%) | n=163 (48·1%) |  |
| Age [median (IQR)] years | 32·9 (28·9; 37·2) | 33·5 (28·9; 38·3) | 32·1 (28·9; 36·8) | 0·082 |
| HIV infected, n (%) | 69 (20·4) | **45 (25·6)** | **24 (14·7)** | **0·013** |
| Smoker, n (%) | 124 (36·6) | **50 (28·4)** | **74 (45·4)** | **<0·001** |
| Asthma/COPD, n(%) | 10 (3·0) | 5 (2·8) | 5 (3·1) | 0·902 |
| Diabetes, n(%) | 2 (0·6) | 1 (0·6) | 1 (0·6) | 0·731 |
| Weight [median (IQR)] | 72·6 (59·1; 89·9) | 72·6 (62·0; 91·5) | 72·8 (55·5; 88·8) | 0·075 |
| **Socio-demographic characteristics** | | |  |  |
| Marital status – single, n(%) | 188 (55·5) | 92 (52·2) | 96 (58·9) | 0·220 |
| Married/cohabiting, n(%) | 151 (44·5) | 84 (47·7) | 67 (41·1) |  |
| Maternal education, n(%) |  |  |  |  |
| Primary education | 21 (6·2) | 9 (5·1) | 12 (7·4) | 0·579 |
| Some secondary education | 190 (56·1) | 95 (54·0) | 95 (58·3) |  |
| Completed secondary education | 115 (33·9) | 65 (36·9) | 50 (30·7) |  |
| Tertiary education, n(%) | 13 (3·8) | 7 (4·0) | 6 (3·6) |  |
| Maternal employment, n(%) | 131 (38·6) | 72 (40·9) | 59 (36·2) | 0·373 |
| Household income per month, n(%) |  |  |  |  |
| <1000 ZAR (<60 USD) | 56 (16·5) | 25 (14·2) | 31 (19·0) | 0·345 |
| 1000-5000 ZAR (60-300 USD) | 221 (65·2) | 115 (65·3) | 106 (65·0) |  |
| >5000 ZAR (>300 USD) | 62 (18·3) | 36 (20·5) | 26 (16·0) |  |
| Number of household members [median (IQR)] | 5 (4; 6) | 5 (4; 6) | 5 (4; 6) | 0·282 |

Abbreviations: CoV2-S spike, IQR=interquartile range; ZAR= South African Rand; USD= United States dollar

**Table S2. Multivariate analysis of factors associated with seropositivity^*^**

|  | **Unadjusted OR (95% CI)** | **Adjusted OR (95% CI)**** |
| --- | --- | --- |
|  |  |  |
| Wave | **3·06 (2·61; 3·59)** | **3·22 (2·71; 3·82)** |
| Age | **1·05 (1·02; 1·08)** | 1·02 (0·98; 1·06) |
| HIV infection | **1·68 (1·06; 2·66)** | 1·63 (0·92; 2·89) |
| Current smoking | **0·50 (0·36; 0·70)** | **0·43 (0·28; 0·66)** |
| Asthma | 0·78 (0·33; 1·85) | 0·77 (0·28; 2·15) |
| Weight | **1·01 (1·00; 1·02)** | 1·00 (0·99; 1·01) |
| Marital status – single | Reference | Reference |
| Married/cohabiting | 1·06 (0·76; 1·49) | 1·03 (0·67; 1·59) |
| Maternal education |  |  |
| Primary education | Reference | Reference |
| Some secondary education | 1·05 (0·53; 2·07) | 1·30 (0·58; 2·94) |
| Completed secondary education | 1·16 (0·57; 2·35) | 1·58 (0·66; 3·76) |
| Tertiary education | 0·89 (0·32; 2·25) | 1·05 (0·26; 4·24) |
| Maternal employment | 1·19 (0·84; 1·68) | 0·93 (0·59; 1·46) |
| Household income per month |  |  |
| <1000 ZAR (<60 USD) | Reference | Reference |
| 1000-5000 ZAR (60-300 USD) | 1·48 (0·96; 2·28) | 1·05 (0·57; 1·93) |
| >5000 ZAR (>300USD) | 1·60 (0·92; 2·80) | 1·61 (0·76; 3·43) |
| Household size | 1·08 (0·99; 1·18) | **1·14 (1·02; 1·27)** |

OR=Odds ratio; CI=confidence interval

*Seropositive defined as S-antibodies to ancestral virus > 1·09 WHO BAU/ml

**339 women included, with 1105 observations; observations after vaccination excluded from analysis

| Variant IgG assessed | Probability of increased titres at minimal pre-wave antibody levels (%, 95% CrI) | Probability of increased titres at maximal pre-wave antibody levels (%, 95% CrI) | 50% reduction threshold (WHO BAU/ml, median, 95% CrI) | N | N increased | Doses pre and post-wave | Proportion of seropositives with pre-wave antibody titres higher than threshold (median) | Proportion of seropositives with pre-wave antibody titres higher than threshold (2·5% CrI) | Proportion of seropositives with pre-wave antibody titres higher than threshold (97·5% CrI) |
| --- | --- | --- | --- | --- | --- | --- | --- | --- | --- |
| Beta | 51·4 (43·2, 77·2) | 19·9 (1·6, 30·7) | 6·3 (1·3, 31·0) | 337 | 134 | 0 | 75·8% (119) | 42·0% (66) | 98·7% (155) |
|  |  |  |  |  |  | Total | 75·5% (120) | 42·1% (67) | 98·7% (157) |
| Delta | 76·2 (69·2, 82·8) | 42·3 (19·9, 63·9) | 36·9 (15·3, 159·5) | 232 | 164 | 0 | 23·0% (35) | 4·6% (7) | 49·3% (75) |
|  |  |  |  |  |  | Total | 24·2% (54) | 3·6% (8) | 52·5% (117) |
| Omicron | 86·3 (79·5, 92·0) | 13·1 (3·7, 25·7) | 185·0 (117·2, 278·6) | 217 | 142 | 0 | 10·5% (15) | 5·6% (8) | 14·7% (21) |
|  |  |  |  |  |  | 1 | 69·1% (38) | 61·8% (34) | 74·5% (41) |
|  |  |  |  |  |  | 2 | 62·5% (10) | 62·5% (10) | 68·8% (11) |
|  |  |  |  |  |  | Total | 28·0% (79) | 24·1% (68) | 32·6% (92) |

**Table S3· Estimated levels of protection for minimal and maximal pre-wave variant-specific antibody titres, 50% protection against seroconversion antibody titre threshold, and proportion of individuals with pre-wave titres above threshold.**

**Table S4· Vaccinations and serostatus prior to vaccine**

|  | **number of vaccines (n=154, %)** | **Seropositive prior to vaccine (n=135, %)** | **Median days between vaccine and blood sample (IQR)** |
| --- | --- | --- | --- |
| **Vaccine** |  |  |  |
| Ad26.COV.2.S | 27 (17·4) | 26 (19·3) | 64 (33; 113) |
| BNT162b2– 1 dose only | 61 (39·6) | 51 (37·8) | 32 (14; 56) |
| BNT162b2– 2 doses | 66 (42·6) | 58 (43·0) | 75 (39; 97·5) |

IQR=Interquartile range

**Table S5 GMCs of participants after 1 or 2 doses of BNT162b2 (Pfizer-BioNTech)** **vaccine stratified by serostatus prior to vaccination**

|  | **One dose (n=72)** | | **Two doses (n=65)** | |
| --- | --- | --- | --- | --- |
|  | **Seronegative prior to vaccine (n=12)** | **Seropositive prior to vaccine (n=60)** | **Seronegative prior to vaccine (n=8)** | **Seropositive prior to vaccine (n=57)** |
|  | **GMC (95% CI)** | **GMC (95% CI)** | **GMC (95% CI)** | **GMC (95% CI)** |
| S-ancestral | 517·92 (190·41; 1408·79)^1^ | 1590·14 (1231·33; 2053·50)^1^ | 819·71 (239·97; 2800·00)^5^ | 1488·78 (1109·50; 1997·70)^5^ |
| S-beta | 348·22 (143·05; 847·67·25)^2^ | 1181·93 (865·85; 1613·38)^2^ | 499·16 (125·69; 1982·26)^6^ | 967·92 (721·63; 1298·28)^6^ |
| S-delta | 393·38 (153·45; 1008·46)^3^ | 1139·07 (852·57; 1521·84)^3^ | 609·91 (162·73; 2286·00)^7^ | 1012·06 (753·54; 1359·28)^7^ |
| S-omicron | 142·15 (46·01; 439·20)^4^ | 469·98 (353·07; 625·60)^4^ | 233·02 (52·50; 1034·25)^8^ | 427·42 (307·18; 594·72)^8^ |

GMC= Geometric mean concentration; CI= confidence interval

^1^ Comparison of GMC S-ancestral titres of 1 dose in seropositive vs seronegative participants, p=0·018

^2^ Comparison of GMC S-beta titres of 1 dose in seropositive vs seronegative participants, p=0·008

^3^ Comparison of GMC S-delta titres of 1 dose in seropositive vs seronegative participants, p=0·026

^4^ Comparison of GMC S-omicron titres of 1 dose in seropositive vs seronegative participants, p=0·007

^5^ Comparison of GMC S-ancestral titres of 2 doses in seropositive vs seronegative participants, p=0·299

^6^ Comparison of GMC S-beta titres of 2 doses in seropositive vs seronegative participants, p=0·299

^7^ Comparison of GMC S-delta titres of 2 doses in seropositive vs seronegative participants, p=0·328

^8^ Comparison of GMC S-omicron titres of 2 doses in seropositive vs seronegative participants, p=0·485

**Figure S1. Flow chart of participants across 4 waves**


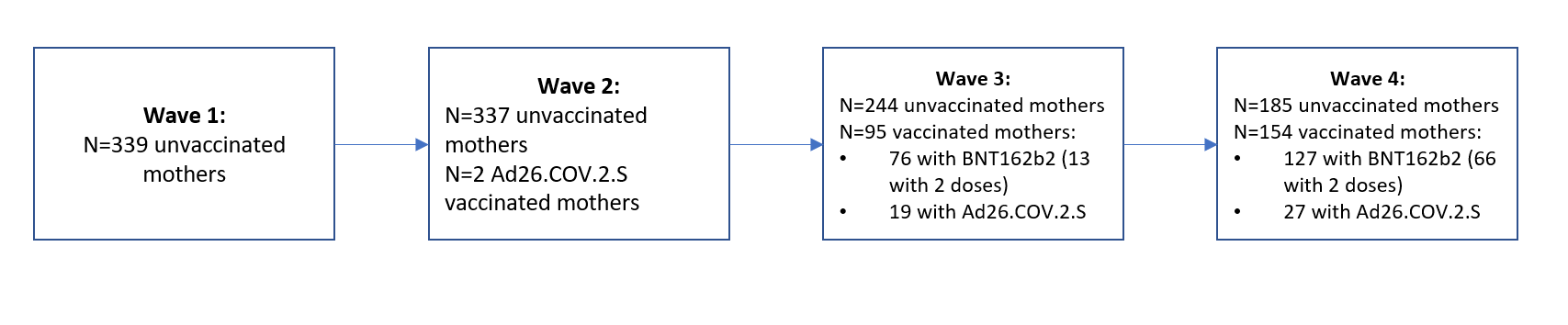


Figure S2: Kaplan Meier curve of Survival as seronegative

**Figure S2. Correlations of wild-type and variant titres, pre and post-wave.**


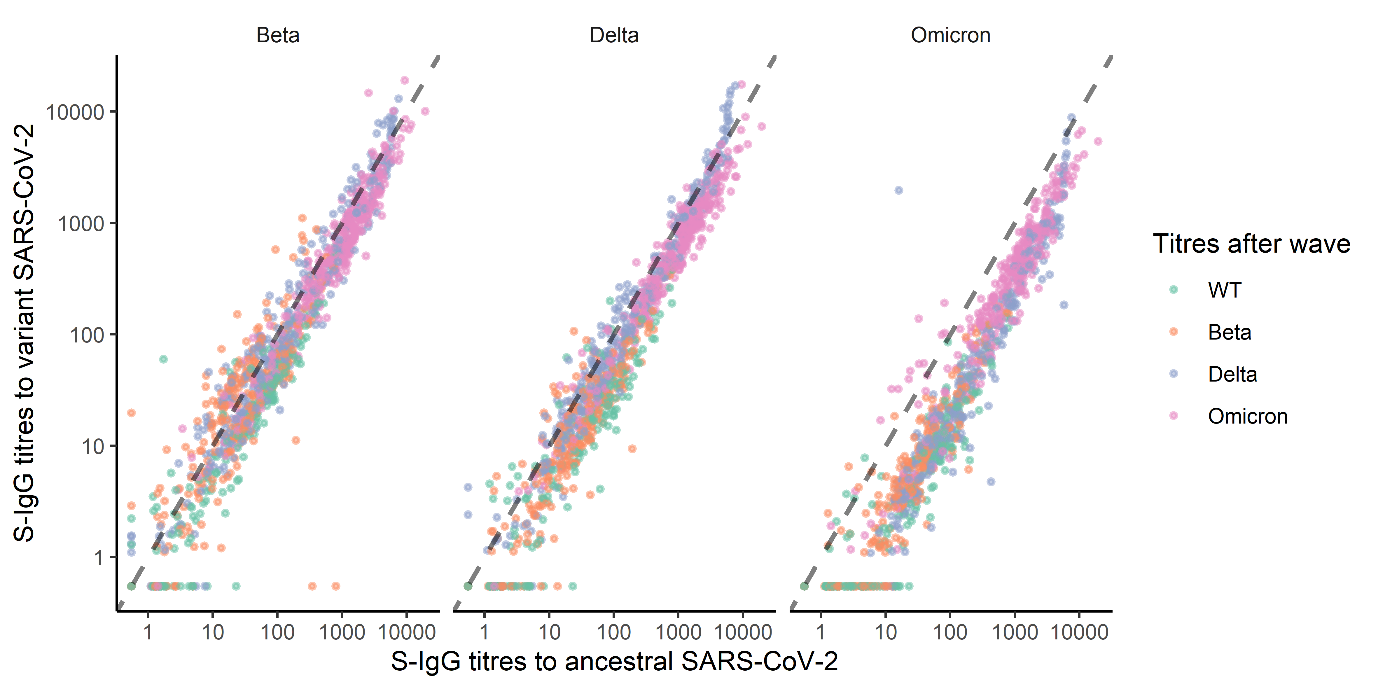


**Model description**

A 4-parameter logistic function was fit to data to estimate the probability of seroconversion (S) at minimal (upper asymptote, C) and maximal (lower asymptote, A) pre-wave IgG titres (log-transformed, titre), as well as the pre-wave IgG threshold required to confer a reduction of 50% in the probability of seroconversion between minimal and maximal protection (the inflection point, m) using Markov Chain Monte Carlo (MCMC) implemented in the Bayesian modelling software JAGS (Just Another Gibbs Sampler, Plummer 2003) via R2jags (Su and Yajima 2021) and R version 4.2.0 (R Core Team 2022).

**
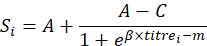
**

**
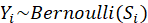
**

Uninformative Gamma and log-normal priors were used for β and m respectively, and weakly informative priors were used for A (Beta(1,2)) and C (Beta(2,1)) to improve identifiability. Models were run for 10,000 iterations with 4 chains, and assessed for convergence visually and via the Rhat statistic.

To test for an effect of vaccination (pooling those who had received either one or two doses) independent of that of IgG-mediated protection in the Omicron wave, an additional term zeta (ζ) was estimated:

**
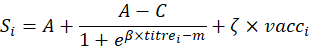
**

**
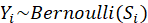
**

Thus ζ represents the % difference in the probability of seroconversion between those who received at least one dose and those unvaccinated, and C-A represents the % difference in the probability of seroconversion between those with minimal and maximal antibody titres. Zeta was then estimated as -8% (95% CrI: -16, 3%) and C-A as -64% (95% CrI: -79, -50%), indicating that having high IgG titres represented the majority of the reduction in the probability of seroconversion rather than some other vaccine-specific factor (immunological or behavioural).

**Sensitivity analysis on waning and choice of threshold**

In order to account for the effect of antibody waning between samples and how this may affect estimates of seroconversion rates and thresholds of protection, we estimated a conservative waning rate using data from those who saw titres decline during the Beta wave (the wave with the lowest estimated attack rate, and prior to the introduction of vaccination) and applied this to the Omicron wave.

The rate of S-IgG waning was estimated by fitting a linear mixed model to S-IgG titres measured before and after the Beta wave in those individuals whose titres declined, against time between samples (in weeks), with a random effect (random intercept) for individual:

$$\log\left( {IgG}_{i} \right)\sim\beta t_{i}+(1|{individual}_{i})$$

Where the rate of waning (ω, percent change per week) is given by $e^{\beta}$-1, yielding a mean waning rate of 3% (95% CI: 2, 4%) per week.

We then calculated an expected post-wave titre given this waning rate and time between pre-and post-wave samples (*t*) for the Omicron wave:

$${expected waned titre}_{i}={{pre wave titre}_{i}\times(1-\omega)}^{t_{i}}$$

The probability of seroconversion in the Omicron wave was then redefined as whether post-wave titres exceeded the expected waned titre, taking the lower bound of ω = 2% per week as a conservative estimate (to avoid spurious seroconversions which amy result from taking the central estimate).

**Table S6 Sensitivity analysis on proportion seroconverted (increased titres) during Omicron wave for different assumptions on thresholds and waning**

| **Assumption** | **Proportion seroconverted during Omicron wave** |
| --- | --- |
| >1% increase on pre-wave (baseline) | 297/418 (71.1%) |
| >10% increase on pre-wave | 297/418 (71.1%) |
| >expected waned titre (2% waning per week) | 330/418 (78.9%) |

There was no difference in the proportion who seroconverted using a 10% compared to a 1% threshold (Table S6). Assuming pre-wave titres waned at a rate of 2% per week resulted in a slightly higher proportion seroconverting during the course of the wave (78.9% vs. 71.1%), which translated to a marginally higher protection threshold (893 WHO BAU/ml (95% CrI: 630, 1451) and a lower proportion protected in the unvaccinated (unvaccinated: 6.7% (95% CrI: 4.3, 11.7%); one dose: 65.5% (95% CrI: 50.0, 69.0%); two dose: 62.5% (95% CrI: 56.2, 62.5%).
